# Possible involvement of self-defense mechanisms in the preferential vulnerability of the striatum in Huntington's disease

**DOI:** 10.3389/fncel.2014.00295

**Published:** 2014-09-26

**Authors:** Laetitia Francelle, Laurie Galvan, Emmanuel Brouillet

**Affiliations:** ^1^Neurodegenerative Disease Laboratory, Commissariat à l'Énergie Atomique et aux Énergies Alternatives, Direction des Sciences du Vivant, Institut d'Imagerie BioMédicale, Molecular Imaging Research CenterFontenay-aux-Roses, France; ^2^Centre National de la Recherche Scientifique - Commissariat à l'Énergie Atomique et aux Énergies Alternatives Unité de Recherche Associée 2210Fontenay-aux-Roses, France; ^3^Intellectual and Developmental Disabilities Research Center, Semel Institute for Neuroscience and Human Behavior, Brain Research Institute, David Geffen School of Medicine, University of California Los AngelesLos Angeles, CA, USA

**Keywords:** striatum, Huntington, markers, cell death, excitotoxicity, signaling, gene products

## Abstract

HD is caused by a mutation in the huntingtin gene that consists in a CAG repeat expansion translated into an abnormal poly-glutamine (polyQ) tract in the huntingtin (Htt) protein. The most striking neuropathological finding in HD is the atrophy of the striatum. The regional expression of mutant Htt (mHtt) is ubiquitous in the brain and cannot explain by itself the preferential vulnerability of the striatum in HD. mHtt has been shown to produce an early defect in transcription, through direct alteration of the function of key regulators of transcription and in addition, more indirectly, as a result of compensatory responses to cellular stress. In this review, we focus on gene products that are preferentially expressed in the striatum and have down- or up-regulated expression in HD and, as such, may play a crucial role in the susceptibility of the striatum to mHtt. Many of these striatal gene products are for a vast majority down-regulated and more rarely increased in HD. Recent research shows that some of these striatal markers have a pro-survival/neuroprotective role in neurons (e.g., MSK1, A2A, and CB1 receptors) whereas others enhance the susceptibility of striatal neurons to mHtt (e.g., Rhes, RGS2, D2 receptors). The down-regulation of these latter proteins may be considered as a potential self-defense mechanism to slow degeneration. For a majority of the striatal gene products that have been identified so far, their function in the striatum is unknown and their modifying effects on mHtt toxicity remain to be experimentally addressed. Focusing on these striatal markers may contribute to a better understanding of HD pathogenesis, and possibly the identification of novel therapeutic targets.

## Introduction

### A summary of what is HD

HD is a dominantly inherited disorder generally affecting young adults. Symptoms include involuntary abnormal movements (chorea, dyskinesia, dystonia), frontal cognitive deficits (e.g., perseveration) and psychiatric disturbances (Harper, 1991; Walker, 2007). The disease is fatal approximately 15 years after the onset of symptoms. There is no treatment available to slow the progression of this devastating disorder.

HD is caused by a mutation in the *HTT* gene encoding the protein huntingtin (Htt) that consists in a CAG triplet repeat expansion translated into an abnormal poly-glutamine (polyQ) tract within the N-terminal region of the protein (The-Huntington's-Disease-Collaborative-Research-Group, 1993). When considering cohorts of HD gene carriers, genetic studies showed that the longer is the CAG repeat expansion the earlier the disease onsets. However, there is a huge inter-individual variability in age of onset (and nature) of symptoms for gene carriers with similar CAG repeat numbers. Thus, apart from HD gene mutation, many genetic, epigenetic and environmental factors may affect the course of the disease (Sturrock and Leavitt, 2010). Deciphering these factors and the underlying mechanisms affecting the onset of this disease could constitute a real hope to find an efficacious treatment to slow the disease.

The mutant protein is cleaved by many proteases leading to the production of N-terminal fragments that form toxic oligomers (Roze et al., 2008b). Eventually mutant Htt (mHtt) forms intranuclear inclusions and somatodendritic aggregates that also contain ubiquitin and represent a histopathological hallmark of HD (Li and Li, 2004a).

Mechanisms of HD pathogenesis have been extensively studied in the past 20 years, since the gene has been identified and cloned. Thanks to many different genetic models (in cells, mice, rat, and even monkeys) a large spectrum of cellular defects has been identified and could contribute to neurodegeneration. For this reason the pathogenesis of HD is often considered multi-factorial. The polyQ expansion in mutated Htt (mHtt) produces a gain-of-function that is toxic to neurons through several mechanisms. One major early event in HD is the alteration of transcription (Cha, 2007; Seredenina and Luthi-Carter, 2012). Importantly, reduced transcription of Brain Derived Neurotrophic Factor (BDNF), a major neurotrophic factor for striatal cells has been found (Zuccato and Cattaneo, 2007). Axonal transport alterations (Li and Li, 2004b; Roze et al., 2008b) leading to several cellular disturbance, including defects in BDNF secretion and transport (Gauthier et al., 2004) also contribute to neurodegeneration. Other alterations include intracellular signaling defects (Borrell-Pages et al., 2006), deregulated of the proteasome pathway (Finkbeiner and Mitra, 2008) and autophagy (Ravikumar and Rubinsztein, 2006), perturbation of calcium homeostasis leading to excitotoxicity (Cowan and Raymond, 2006; Raymond et al., 2011), mitochondrial defects and oxidative stress (Damiano et al., 2010).

In addition, the mutation in one allele is thought to produce a loss of function of wild type Htt (Cattaneo et al., 2005). Indeed, htt is involved in a large variety of physiological cellular processes. It regulates vesicle transport through regulation of molecular motors of the cytoskeleton, transcription of important pro-survival factors (such a BDNF) by interacting with key transcription factors and co-activators of transcription, cell division, intracellular signaling and ATP production (Zuccato and Cattaneo, 2014).

While wild type and mHtt protein are ubiquitously expressed in the brain, degeneration primarily affects the striatum. The contribution of striatal degeneration in motor and cognitive symptoms is not totally understood but neuropathological studies showed that striatal atrophy correlates with severity of symptoms (Myers et al., 1988). Recently, follow up of HD gene carriers cohort using Magnetic Resonance Imaging (MRI) and Positron Emission Tomography (PET) showed that even at presymptomatic stages, the atrophy of the striatum is detectable and may start even 10 years before onset of symptoms (Tabrizi et al., 2013). Other brain regions may also be damaged at early stages, such as the hypothalamus, and at later stages the cerebral cortex and other regions also degenerate (for a review, Brouillet et al., 1999; Petersen and Bjorkqvist, 2006). Thus, HD is not a selective striatal disease. Many innovative studies discovered extra-striatal and peripheral anomalies in HD animal models and for particular studies in HD patients (Martin et al., 2008; Obeso et al., 2014). However, the preferential striatal degeneration is an intriguing characteristic of this illness, and the underlying mechanisms may represent an important aspect of HD pathogenesis.

### Existence of possible compensatory mechanisms in HD

The existence of compensatory mechanisms in HD (as for other neurodegenerative diseases) is probable. Possibly, the best circumstantial evidence for this is that although mHtt is expressed in the brain of HD gene carriers since birth, degeneration and symptoms appear during adulthood (with the exception of long CAG repeat expansion carriers who develop the disease during childhood) (Harper, 1991; Walker, 2007). Similarly in genetic animal models, degeneration and symptoms occur in adult or aged animals (Menalled and Chesselet, 2002; Menalled, 2005). It has been shown that when mHtt is expressed in striatal neurons at similar levels for the same duration, its neurotoxic effects are significantly higher in aged animals, as compared to young animals (Diguet et al., 2009). The reason for this age-dependent phenomenon is unknown but it indicates that neurons possess the ability to partially counteract cellular stress induced by mHtt, a plasticity mechanism that may be progressively lost with aging. The aim of this review is not to cover all the possible compensatory mechanisms that may occur in the HD brain, but to focus on those that can be found in the striatum. However, a few examples of potential compensatory mechanisms that could be encountered in all cell types can be given.

There likely exist compensatory mechanisms at whole human brain level, to overcome cell dysfunction and/or neurodegeneration in the striatum of HD patients. For example, PET studies showed that effective learning performance on motor sequence learning tasks, normally associated with activation of the dorsolateral prefrontal cortex and the caudate nucleus, was not requiring the same brain regions in presymptomatic HD (pre-HD) patients and healthy volunteers (Feigin et al., 2006). In presymptomatic HD gene carriers, ventral prefrontal and orbitofrontal regions were used possibly via thalamic projections.

At cellular level, transient/reversible transcriptional and post-transcriptional mechanisms may intervene to compensate for cell suffering and degeneration pathways. For example, the loss of expression of the kinase PKCδ (Rue et al., 2014) is likely a compensatory mechanism. Indeed, the overexpression of PKCδ enhances mHtt toxicity *in vitro*. On the contrary, the knock down of PKCδ (using siRNA strategy or expression of a dominant negative form) significantly reduces mHtt effects. Interestingly the loss of PKCδ seems to occur through an increased degradation of the protein by neurons expressing mHtt (Rue et al., 2014).

Examples of potential compensatory mechanisms in HD can be found in studies related to defects energy metabolism that are thought to occur early in HD. Unexpectedly, recent experiments show that an early increase in the levels of high energy phosphate metabolites (ATP, phosphocreatine) can be found in the brain of HD mouse models (Mochel et al., 2012a; Tkac et al., 2012). Consistent with these observation in genetic models of HD, dynamic measurements of brain phosphocreatine levels during synaptic activation in HD patients using ^31^P NMR spectroscopy also demonstrate abnormalities in the use of high energy phosphate metabolites (Mochel et al., 2012b). In R6/2 and Knock-in 111Q mouse models, early biochemical changes indicate that neurons tend to compensate by activating energy promoting cellular pathways (Mochel et al., 2012a). In particular, possible compensatory changes occur at the post-translational levels, leading to an increase in AMPK phosphorylation in HD mice, which could activate pathways leading to a more efficient metabolism.

Large scale analyses trying to broadly identify mRNA and/or protein expression changes provide a huge amount of information from which potential compensatory mechanisms in HD may be discovered. A well-controlled proteomic analysis of brain of R6/2 HD mice at different ages underlined that a number of proteins display transient /biphasic expression changes rather than an age-dependent progressive decline (Zabel et al., 2009). For instance, the absolute expression of the mitochondrial complex II subunit Ip (iron-sulfur), a key regulator of oxidative energy metabolism which is neuroprotective against mHtt (Benchoua et al., 2006; Damiano et al., 2013), is early reduced in 2 week-old R6/2 mice, but is found to be increased in 8 weeks old of these mice and brings back at basal levels at 12 weeks old (Zabel et al., 2009).

Changes in the expression levels (decreases and more rarely increases) of mRNA in HD have been extensively explored in the last decade (Seredenina and Luthi-Carter, 2012). These changes may indicate two types of phenomena. On one hand, it indicates primary defects of transcription inherent to the presence of mHtt. In many cases, the direct interaction of mHtt with proteins that are part of macromolecular complexes involved in transcription regulation leads to a reduction of transcription and reduced levels of a large spectrum of gene products (Seredenina and Luthi-Carter, 2012). On the other hand, changes in mRNA levels (or protein) may not be directly linked to a primary effect of mHtt but could rather result from a physiological response engendered by the cellular stress induced by toxic gain of function of mHtt. Many expression changes identified in large scale analyses have been studied with the hypothesis that they were causal in HD pathogenesis. It is not always the case. Expression changes can represent self-defense mechanisms. To differentiate between the two above mentioned mechanisms, knock-down/knock-out or overexpression/neuro-rescue experiments in HD models are needed. It is beyond the scope of the present review to provide a detailed description of the gene products that have been experimentally tested. Here we will limit our review to gene products that have deregulated expression and that are preferentially expressed in the striatum. The review of the studies focused on “striatal gene products” illustrates that in some cases, expression changes may represent compensation or self-defense mechanisms while in others they directly contribute to degeneration of striatal neurons.

### Studying the preferential vulnerability of the striatum to identify potential modifiers

#### Working hypothesis

The particular vulnerability of the striatum in HD likely resides in its molecular complexity. Whether its particular vulnerability depends on only one or a subset of gene products, acting together, is unknown. Recent publications indicate that the experimental knock-down or overexpression of only one striatal gene product can significantly change the toxicity of muHtt in cell models and mouse models. In one instance, a single nucleotide polymorphism in a striatal gene, *ADORA2A* (adenosine receptor 2a) has been found to be associated with earlier onset of symptoms in large cohorts of HD patients (Dhaenens et al., 2009). Thus, striatal gene products can have a significant impact of HD. From a therapeutic point of view, this indicates that acting on one single target may be sufficient to alter the course of the disease. Therefore, trying to decipher the complex mechanisms underlying neurodegeneration in the striatum may help to more broadly highlight important factors of neuronal dysfunction and death, and to point potential therapeutic interventions for HD (Brouillet et al., 2005; Thomas, 2006; Brochier et al., 2008; Mazarei et al., 2010).

The study of these causal or compensatory changes in the striatum in HD may also help to better understand other neurological diseases where the striatum is functionally affected (e.g., Wilson, Parkinson, metabolic diseases, addiction, depression etc.).

#### The notion of striatal markers

The hypothesis that gene products preferentially expressed in the striatum (or more generally particularities of this brain region) could play an important role in the susceptibility of the MSN to mHtt toxicity has been studied for many years. Hypotheses related to particular properties of the MSN related to energy metabolism/oxidative stress, or glutamate –related excitotoxicity, and other types of neurotransmitter systems that could explain striatal atrophy in HD where proposed in the 80's and 90's (for a review, Brouillet et al., 1999). The most recent developments of transcriptomic analysis led to a broader “without *a priori*” approach of the working hypothesis that striatum vulnerability to mHtt could reside in the expression of one or a subset of striatal enriched gene products.

The notion of striatal marker stems on the contrast of expression between the striatum and other brain regions. Relatively old studies identified striatal markers based on studies using *in situ* hybridization, immunohistochemistry, and biochemistry (see references in Desplats et al., 2006, for a number of validated striatal markers). The identification of approximately 50 validated markers took approximately two decades. In-depth transcriptomic analyses using serial analysis of gene expression (SAGE) further characterized the molecular complexity of the striatum as compared with other brain regions in mice allowed for the identification of a large list of “striatal markers” in wild type mice (de Chaldee et al., 2003; Brochier et al., 2008; Mazarei et al., 2010). This approach, based on the collection of polyA-containing RNA, provided a ranking of the number of copies of the different RNA species in different regions in the mouse brain. Comparison between brain regions led to the identification of gene products whose expression shows high enrichment in the striatum. Known striatal markers were found, but many annotated gene products whose function in the striatum is unknown were also identified. Approximately, 100–150 striatal markers can be listed, many of which have been cross-validated in different studies (de Chaldee et al., 2003; Desplats et al., 2006; Brochier et al., 2008; Mazarei et al., 2010). Transcriptomic studies using oligonucleotide array or RT-PCR showed that the magnitude of transcriptional changes in the striatum of HD mouse models for these genes preferentially expressed in the striatum was higher than that of ubiquitously expressed genes (Desplats et al., 2006). In the SAGE studies by Brochier and collaborators (Brochier et al., 2008), a number of gene products of unknown neurobiological function showed reduced expression in the striatum of R6/2 HD mice. Transcriptomic DNA array data in HD models and HD brain show that amongst the RNAs whose expression is deregulated, those coding for striatal markers are proportionally more frequently altered (Hodges et al., 2006; Kuhn et al., 2007). Another study validated a number of these striatal markers and identified potentially new ones that were found to be deregulated in YAC128 HD mice (Mazarei et al., 2010). Supplemental Table 1 indicates the striatal markers that have been well validated based on the studies quoted above.

Thus, the notion of striatal marker has evolved with the progression of the analytical methods. The criteria to decide whether a gene product is “preferentially” expressed in the striatum remains debatable. In most cases, the currently available public databases (Allen Brain Atlas) providing gene products expression in the brain in mice and humans generally confirm that the “striatal markers” identified in the studies described above have preferential striatal expression. In general, the contrast of “striatal specificity” in comparison to the somatosensory and motor cerebral cortex is in the range of 3–10-fold enrichment. If we were to consider a lower contrast (a two-fold difference between cortex and striatum for example), the list of striatal markers would be much longer. In addition, it must be mentioned that some striatal gene products, although referenced as “striatal markers” can have stronger expression in other anatomically restricted brain regions such as the hippocampus or some thalamic nuclei.

This review aims at providing a concise overview of the striatal markers that have been experimentally assessed for their capacity to modify mHtt toxicity. These markers have a large spectrum of biological functions and the alteration of the expression levels in HD is not *a priori* indicative of their role in striatal vulnerability. The different striatal gene products that have been experimentally studied for their capacity to change mHtt toxicity can be classified as “protoxic,” “neuroprotective,” and “neutral.” In some instances, the expression changes (up or down) suggest the existence of a compensatory “self-defense” mechanism. We will also point to the large list of the other striatal markers that remain to be fully investigated to determine their potential role in HD.

### Potential protoxic striatal gene products

#### D2-R (Dopamine type 2 receptor)

The hypothesis that dopamine, which is at high concentrations in the striatum compared to other brain areas, might play an important role in the preferential vulnerability of the striatum in HD has been suggested long time ago (Reynolds et al., 1998; Jakel and Maragos, 2000).

Anatomically, MSNs expressing D2-R (D2 MSN) receive preferentially inputs from the Pyramidal Track type (PT-type) cortical neurons whose projects ipsilaterally to the striatum. This preferential innervation is believed to release more glutamate which could contribute to make D2 MSNs more vulnerable to excitotoxicity (Reiner et al., 2003; Ballion et al., 2008). Many electrophysiological evidences suggest that D2 MSNs are more excitable than D1 MSNs (Cepeda et al., 2007; Kreitzer and Malenka, 2007) partly because they display fewer primary dendrites (Gertler et al., 2008). Electrophysiological recordings of D2 MSNs show a higher frequency of spontaneous excitatory post-synaptic currents (sEPSCs) than direct pathway. Moreover, D2 MSNs display large membrane depolarizations rarely seen in direct pathway MSNs (Cepeda et al., 2008) after the addition of GABA_A_ receptor blockers inducing epileptic form activity in CPN (Galvan et al., 2012a). Taken together, these evidences support the idea that D2-MSN is a fertile ground to develop abnormal responses.

Studies performed in YAC128 HD mouse model conducted at a presymptomatic age (1.5 months) and at symptomatic age (12 months) revealed interesting findings concerning the indirect pathways. At presymptomatic age, no differences were observed in excitatory and inhibitory synaptic transmission compared to WT. When the animals are symptomatic and become resistant to excitotoxicity, the inhibitory transmission in YAC128 D2 MSNs is greatly increased (Andre et al., 2011). This may indicate that the indirect pathway is subject to compensatory mechanism in HD, resulting in turn to the slowdown of excitatory glutamatergic synapses in the striatum.

Whether these changes in D2 MSN are only related to D2-R signaling is not known. Direct support for a causal role for DA and D2-R in HD comes from the recent demonstration that the toxicity of the N-terminal fragments of mHtt is potentiated by dopamine in cells expressing mHtt exon 1 and transgenic HD mouse models (Charvin et al., 2005; Cyr et al., 2006; Stack et al., 2007; Benchoua et al., 2008). Dopamine modifies the formation of Htt-containing aggregates in primary striatal neurons transfected with exon 1 of Htt gene and exacerbates mHtt-induced cell death (Charvin et al., 2005). Of interest, this effect involves D2-R signaling, since dopamine effect is blocked by D2 antagonists (Charvin et al., 2005; Benchoua et al., 2006). Dopamine loses its detrimental effect when neurons are prepared from D2 receptor null mice (Charvin et al., 2005). Chronic blockade of the D2-R with a selective antagonist significantly reduces death of MSN in a lentiviral model of mHtt expression in rats (Charvin et al., 2008). Possibly, this “protoxic” effect of dopamine through D2-R stimulation may involve a reduction of the mitochondrial complex II, a key regulator of energy metabolism in neurons (Benchoua et al., 2008). D2-R stimulation increases mHtt toxicity in mouse striatal neurons via, among others, the activation of JNK pathway and activation of the Rho/ROCK-II pathway (Charvin et al., 2005; Deyts et al., 2009).

Thus, the presence of D2-R on MSN may render these neurons more susceptible to HD. However, expression of these receptors is down regulated early in HD as seen using biochemical experiments and PET scans in patients (Antonini et al., 1998; Glass et al., 2000). Whether this decrease is entirely caused by a direct regulation of D2-R transcription by mHtt is unknown. It is conceivable that this decrease is, at least in part, an attempt of MSN to reduce cellular stress generated by mHtt.

#### D1-R (Dopamine type 1 receptor)

In line with a role of D2-R, D1-R may also be involved in the vulnerability of the striatum. Stimulation of D1-R promotes the aggregation of N-terminal fragments of mHtt and cell death in cell line in culture (Robinson et al., 2008). The mechanisms are unknown but a protoxic role for D1-R has been suggested to be mediated by regulation of glutamatergic synapse and facilitation of excitotoxicity (Tang et al., 2007). Supporting this view, experiments in cells immortalized from knock-in HD mice (111Q) showed that activation of D1-R exacerbates mHtt–induced cell death (Paoletti et al., 2008). D1-R activation facilitates glutamate receptor-mediated activation of the Ca^2+^-dependent protease calpain that in turn cleaves Cyclin dependent kinase 5 (Cdk5). Cleavage of Cdk5 activator p35 into p25 would be neurotoxic to striatal neurons (Paoletti et al., 2008). As for D2-R, D1-R expression being reduced in HD patients and HD models, this may also be seen as a self-defense mechanism to reduce mHtt toxicity.

#### CalDAG-GEFI (a.k.a. RASGRP2, calcium and DAG-regulated guanine nucleotide exchange factor I)

CalDAG-GEFI is a guanine-nucleotide exchange factors (GEFs) activated by diacylglycerol (DAG) and Ca^2+^. CalDAG-GEFI has substrate specificity for Rap1A, and was found to be enriched in the basal ganglia (Kawasaki et al., 1998). This striatal gene product has been rarely studied, and its neurobiological function is not totally understood.

A pioneering study showed that expression of this gene product may render striatal cells more vulnerable to mHtt (Crittenden et al., 2010). Interesting, it was shown that striatal neurons of R6/2 mice with the highest level of mHtt-containing aggregates had the lowest levels of CalDAG-GEF. Since macroscopic aggregates are thought to be neuroprotective since they sequester mHtt toxic soluble oligomeric species, these results indicated that the presence of high levels of CalDAG-GEF may lead to increased levels of toxic species of mHtt in transgenic mice. Supporting this view, knock-down of CalDAG-GEF in a brain slice model of HD is neuroprotective against mHtt-induced neurodegeneration. The mechanisms underlying its “pro-toxic” properties are not determined. One possibility is that it may inhibit Ras-dependent activation of the Erk/MAP kinase cascade in striatal neurons. Thus, its diminished expression in HD may allow “re-activation” of the pro-survival Erk/MAP kinase pathway to block mHtt toxicity (Crittenden et al., 2010).

#### RGS2 (Regulator of G-protein signaling 2)

The RGS2 protein is a member of the RGS family of proteins that binds Gα subunits of heterotrimeric G proteins. RGS2 interfere with Gαq and Gαi to reduce their rate of hydrolysis of GTP to GDP and thus inhibits the signal transduction from GPCRs. RGS2 play a key role in synaptic plasticity (Kehrl and Sinnarajah, 2002). RGS2 directly interacts with adenylyl cyclases to inhibit the production of cAMP. RGS2 may also regulate GPCR-mediated Akt signaling (Anger et al., 2007). RGS2 expression is reduced in the HD brain and HD mouse models. Seredinina and collaborators studied whether the loss of RGS2 could exacerbate or reduce neurodegeneration induced by overexpression of mHtt in striatal neurons using lentiviral vectors (Seredenina et al., 2011). Results showed that increased expression of RGS2 further aggravates mHtt-induced neurodegeneration. Underlying mechanisms of RGS2 protoxic effects are not fully deciphered but the authors provided preliminary data indicating that they may implicate regulation of Erk/MAP kinase signaling.

#### Rhes (a.k.a. RASD2, Ras homolog enriched in striatum)

Rhes is a small G-protein that displays striking enrichment in the striatum and can regulate signaling through G-protein coupled receptors (Falk et al., 1999; Vargiu et al., 2004; Mealer et al., 2014). It has been described as a mediator of mHtt cytotoxicity (Subramaniam et al., 2009), acting as a regulator of SUMOylation. The presence of Rhes in MSN would favor the accumulation of toxic oligomeric species of mHtt in the cytoplasm. More recently, the deletion of Rhes has been found neuroprotective in HD R6/1 mice (Baiamonte et al., 2013).

Rhes binds Beclin-1 and activates autophagy, a lysosomal degradation pathway critical in aging and neurodegeneration (Mealer et al., 2014). Activation of autophagy has been shown to be neuroprotective in HD models (Ravikumar and Rubinsztein, 2006). Rhes-induced autophagy is inhibited by mHtt. The restricted expression of Rhes and its effect on autophagy may explain the selective striatal pathology and delayed onset of HD.

#### DGK (Diacylglycerol kinase)

The expression of DGK is increased in the striatum of R6/2 HD mice. Zhang and collaborators deciphered the potential role that this increase may have in striatal degeneration/dysfunction after having identified this kinase as a potential therapeutic target based on a screening of kinase inhibitors in a cellular models expressing mHtt (Zhang et al., 2012). The inhibitor of DGK (R59949) blocked induction of cell death pathways triggered by serum withdrawal in knock-in (111Q/111Q) HD striatal cells. Knockdown of all isoforms of DGK using siRNA strategy demonstrated that selective inhibition of DGKε was responsible for the neuroprotective effect of the inhibitor. Zhang and collaborators found that knocking down DGK gene in a fly model of HD was neuroprotective. Altogether these data indicate that increased DGK in the striatum could contribute to striatal degeneration. DGK increase could be considered as a protoxic event in HD pathogenesis.

#### Calcineurin (or protein phosphatase 3, formerly known as protein phosphatase 2B)

Since 1986, calcineurin has been identified by Goto as a marker of neuronal degeneration in the striatum of HD patients (Goto et al., 1986). Calcineurin has preferential expression in the striatum and is downregulated in HD patients and mouse models of HD (Xifro et al., 2009). Calcineurin dephosphorylates Htt at serine 421, inhibition of calcineurin restores axonal transport and transport of BDNF vesicles (Pineda et al., 2009). It is known that Htt phosphorylation is an important protective mechanism in striatal neurons (Humbert et al., 2002). Phosphorylation of mHtt at serine 421 promotes neuroprotection in HD, by restoring Htt function and the transport of BDNF. Supporting the view that reduced calcineurin may be neuroprotective in HD, increased Htt phosphorylation can be produced by pharmacological inhibition of calcineurin with the immunosuppressor FK506 (also known as tacrolimus and fujimycine) (Pardo et al., 2006), or by overexpression of the regulators of calcineurin RCAN1-1L (Ermak et al., 2009) leading to neuroprotective effects.

Thus, the reduction of calcineurin expression and function would lead to a diminution of its activity, increasing phosphorylated state of key proteins, especially mHtt at S421, that activate survival pathways. These mechanisms may be regarded as a compensatory phenomenon that could retard the progression of striatal degeneration.

#### PDE1B and PDE10A (Phosphodiesterase 1B and 10A)

Studies on phosphodiesterase (PDE) in HD models have shown preferential reduction of the isoforms PDE1B and PDE10A in HD models, while expression of other PDEs seems relatively maintained (Hebb et al., 2004). The loss is detected before onset of symptoms in R6/2 and R6/1 models. Because, PDE regulates levels of cAMP, which plays a key role in modulation of gene expression which is altered in HD, the effects of a treatment with a PDE10 inhibitor has been studied in the R6/2 mouse model of HD. Results showed that chronic pharmacological blockade of PDE10 is neuroprotective and reverses a number of transcriptomic anomalies in HD mice (Giampa et al., 2010). In line with this, the characterization of the effects of a pharmacological inhibition of PDE indirectly suggests that the reduction of PDE activity in HD could lead to multiple effects: it up-regulates cAMP-responsive element –dependent transcription, it down-regulates HDAC4 (histone deacetylase 4) mRNA, and could activate Mitogen- and stress-activated kinase-1 (MSK1). These latter effects should contribute to striatal neurons against mHtt toxicity. Thus, the presence of PDE in striatal cells may be considered protoxic, and its decrease in HD could be seen as a compensatory mechanism to counteract the effect of mHtt. Interestingly, further inhibition of the enzyme may allow the triggering of neuroprotective pathway and as such may constitute an interesting pharmacological therapy.

### Potential neuroprotective striatal gene products

#### BCL11 (B-cell leukemia/lymphoma 11B)

B-cell leukemia/lymphoma 11B (Bcl11b) (a.k.a. CTIP2) is a transcription factor that has been described to be a key gene for differentiation of medium sized spiny neurons in the striatum. Since MSN represent ~95% of the neurons in the striatum, Bcl11b likely possesses a central role that determines the architecture and organization of the striatum, and as such its function is likely crucial in HD (Arlotta et al., 2008). Bcl11b mRNA levels are reduced in the HD striatum. The overexpression of Bcl11b has been found neuroprotective in cell models of HD *in vitro* (Desplats et al., 2008). The direct interaction of Bcl11b with mHtt and its possible sequestration in inclusions may further abolish its capacity to regulate the expression of many striatal genes that are crucial for the survival of MSN. In particular, there exists a functional interaction between Bcl11b and BDNF. Chromatin-immunoprecipitation experiment and sequencing (ChIP-seq) indicated that Bcl11b is a regulator of the BDNF signaling pathway (Tang et al., 2011). Thus, the loss of Bcl11b in the striatum may lead to a striatal-selective cascade of events that could explain the preferential vulnerability of MSNs against mHtt.

#### FOXP1 (Forkhead box protein P1)

FOXP1 is thought to be an important transcription factor regulating cell-cell interaction signaling. FOXP1 shows highly expression in the striatum (Desplats et al., 2006, 2008). Its expression is regulated by Bcl11b. There exist overlaps between the genes that are regulated by FOXP1 in normal neurons and the genes that are deregulated in HD (Tang et al., 2012). No rescue or knock-down experiments have been performed, but FOXP1 seems to interact with mHtt and to be trapped in mHtt-containing aggregates (Tang et al., 2012). Therefore, its reduced expression likely contributes to the preferential vulnerability of the striatum in HD.

#### MSK-1 (Mitogen- and stress-activated kinase-1)

In healthy conditions, the mitogen- and stress-activated kinase-1 (MSK-1), a striatum-enriched nuclear protein kinase downstream Extracellular Regulated Kinase (ERK), promotes activation of the transcriptional factor kappa-light-chain-enhancer of activated B cells (NF-kappaB) signaling, inducing c-Fos transcriptional activation important for immune and inflammatory responses (Vermeulen et al., 2003). MSK-1 is downregulated in R6/2 HD model mice and in caudate from HD patients (Roze et al., 2008a). Overexpression of MSK-1 in primary culture of striatal neurons expressing a short fragment of mHtt is neuroprotective, whereas knockdown of MSK-1 is protoxic. Interestingly Roze and collaborators found evidence of ERK, Elk-1, and CREB nuclear activation in the striatum of R6/2 mice. This suggested the existence of a possible self-defense response in striatal neurons. However, this response appeared to be blunted, since neither phosphorylation of histone H3 phosphorylation nor c-Fos activation were detected. Indeed, loss of MSK-1 in the striatum in HD mice impeaches activated ERK to produce its downstream effects on transcription. In the normal brain, MSK-1 phosphorylates histone H3, CREB and up-regulates peroxisome proliferator-activated receptor co-activator-1α (PGC-1α), playing role in bioenergetic stability in MSNs. The MSK-1 downregulation likely produces mitochondrial dysfunction rendering MSNs more susceptible to mHtt. Consistent with this hypothesis, MSK-1 overexpression in striatal neurons using lentiviral vectors was neuroprotective against mHtt in mouse models of HD (Martin et al., 2011). Therefore, because MSK-1 shows enrichment in the striatum, its loss would contribute to render the striatum more fragile in HD.

#### ADORA2 (Adenosine receptor type 2A)

A2A receptors (A2A-R), coded by the *ADORA2A* gene have a highly enriched expression in the striatum. The expression of A2A receptor is down regulated in the striatum of HD patients (Glass et al., 2000) and in several HD mouse models (R6/2,N171-82Q) (Menalled et al., 2000; Chou et al., 2005) These receptors are located at the terminal of cortico-striatal pathway (presynaptic receptors) and in the D2-MSNs (post-synaptic receptors). The mRNA level of A2A-R in the striatum is higher in the striatum than in the cerebral cortex. These two types (pre- and post-synaptic) seem to differ in their contribution to neurodegenerative process. Evidences in HD area suggest that activation of presynaptic A2A-R is pro-toxic for MSNs by modulation of glutamate release whereas activation of post-synaptic A2A-R are protective (Popoli et al., 2007). Both agonists and antagonists were proposed to treat HD symptoms. Interestingly, the A2A-R agonist, CGS21680, produces an opposite effect in WT and symptomatic R6/2 in slices. Field potentials (FP) were recorded with and without NMDA and CGS21680. The NMDA toxicity is observed by the only partial recovery after the FP stimulation. The addition of CGS21680 increases NMDA-mediated toxicity in WT MSNs whereas it decreases it in symptomatic R6/2 mice (Martire et al., 2007). Thus, it seems that complex regulatory mechanisms, possibly compensatory, involve A2A-R in HD mice.

The chronic effect of the presence of A2A-R, especially expressed at high level in MSN is not totally understood. Genetic deletion of the *ADORA2A* gene precipitates motor symptoms and death in HD mice expressing a short N-terminal fragment of mHtt (Mievis et al., 2011a). In support of the hypothesis that A2A-R may have an impact on the disease progression, a single genetic polymorphism in the *ADORA2A* gene in HD patient can modify the age of onset (Dhaenens et al., 2009). Thus, the loss of A2A-R may be detrimental. These receptors are likely neuroprotective. However, it must be underscored that the exact contribution of presynaptic receptors of the cortico-striatal pathway vs. the post-synaptic receptors expressed by MSN in these experiments remains to be fully elucidated.

#### CNR1 (Cannabinoid type 1 receptor)

The profound and early loss of striatal type 1 cannabinoid receptors (CB1-R) in the striatum and projection area (substantia nigra reticulate and globus pallidus externus) in HD has been demonstrated by autoradiography studies on post-mortem brain samples from patients at early stages as for the A2A-R (Glass et al., 2000). Loss of CB1 binding sites have been confirmed *in vivo* by PET studies in HD patients (Van Laere et al., 2010). Elegant studies demonstrated that genetic deletion/knockout of CB1 receptors exacerbates the motor phenotype in HD mice (Blazquez et al., 2011; Mievis et al., 2011b). The loss of CB1-R might be due to direct transcriptional deregulation produced by mHtt (via mHtt-induced deregulation of REST) (Blazquez et al., 2011) but also may result from more complex mechanisms. Indeed, exposure of immortalized striatal cells with endogenous cannabinoids produced an increase in CB1-R expression (Laprairie et al., 2013). Treatment of HD striatal cells (Q111/Q111) with cannabinoid markedly increases CB1-R expression. Available results from *in vitro* experiments indicate that the loss of CB1-R in HD would lead to reduced levels of BDNF, which in turn should render striatal cells more vulnerable to mHtt toxicity, possibly through decreases in PGC-1α levels (Laprairie et al., 2013). However, a convincing work recently performed in R6/2 HD mice showed that only the presynaptic CB1-R at the cortico-striatal terminals actually underlie the neuroprotective effects of the CB1-R agonists *in vivo* (Chiarlone et al., 2014). Thus, CB1-R can be considered as neuroprotective. However, the impact of the reduced striatal expression of CB1-R in HD is uncertain.

#### SCN4b (Sodium channel beta 4b subunit)

SCN4b mRNA expression is down regulated in HD models and HD patients (Oyama et al., 2006; Kuhn et al., 2007; Brochier et al., 2008). Its reduced expression is more severe than that of other sodium channel subunits (Oyama et al., 2006). The function of this sodium channel subunit is unknown. The good correlation between loss of its expression and progression of the disease in R6/2 mice suggested a potential role in striatal vulnerability. In line with this, SCN4b levels seem to be more reduced in regions of the central nervous system that are the most affected by mHtt expression. Interestingly, overexpression of SCN4b in neurons in primary culture produces trophic effects characterized by increased dendritic genesis (Oyama et al., 2006). Thus, SCN4b may be a “neuroprotective” striatal marker whose reduced expression in HD may contribute to the preferential degeneration of the striatal in HD. However, its putative neuroprotective effect needs to be directly assessed against mHtt toxicity.

#### STEP61(PTPN5 gene, striatal-enriched protein tyrosine phosphatase 61)

Reduced expression of STEP61 mRNA has been found in HD transgenic models and HD brain (Desplats et al., 2006). In different mouse models (YAC1128, TET-HDH94, R6/1, and KI111) the protein is reduced and its level of phosphorylation is increased, which should further contribute to a reduction of its phosphatase activity (Saavedra et al., 2011; Gladding et al., 2014). Convincing results indicate that the loss of STEP61 is globally detrimental to MSN, although it may also partially represent a compensatory mechanism trying to block excitotoxicity in striatal cells. In R6/1 mice, whereas STEP protein levels are reduced in young (excitotoxicity sensitive) mice, its levels of phosphorylation is much increased, leading to its further inactivation (Saavedra et al., 2011). In line with this, intrastriatal injection of a permeable and active form of SETP61 (TAT-STEP), could increase the excitotoxic lesions produced by the NMDA receptor agonist quinolinate. In addition, an increased cleavage of STEP61 has been observed, resulting from increased calpain activation due to entry of Ca^2+^ through NMDA receptors. An accumulation of the breakdown product STEP33 (inactive and unable to dephosphorylate MAPK/p38) is associated with elevated p38 phosphorylation (Saavedra et al., 2011), which his detrimental for cell survival. STEP dephosphorylates ERK, reducing its activation and pro-survival signals. There is an increased activation of pro-survival MAPK/ERK1/2 signaling in older mice resistant to excitotoxicity. In young YAC128CAG HD mice that are sensitive to excitotoxicity, STEP61 levels have also been found reduced, as STEP33 (Gladding et al., 2014). At later stage, when YAC128 mice become resistant to excitotoxicity, the loss of STEP61 may be associated with the induction of ERK1 (blocking excitotoxicity) while maintaining activation of MAPK/p38 that favors cell death pathways. These very interesting studies clearly show the existence of complex “striatum-specific” compensatory mechanisms in HD mice, and their evolution over time, possibly to block sequentially mHtt toxicity.

Thus, the role of STEP61 in striatal vulnerability is ambivalent. Its loss in HD may reduce excitotoxicity, consistent with a neuroprotective compensatory mechanism. In this case STEP61 could be considered as a protoxic actor in MSN. However, its loss also contributes to activate MAPK/p38 pathway. In this latter case, STEP61 may be seen as a neuroprotective agent for MSNs.

#### ELK-1 (ETS-like gene 1)

In basal condition, Elk-1 is ubiquitously expressed in the brain, but in HD mice models R6/1 and R6/2, and in immortalized HD mouse (Q111/Q111) cells, Elk-1 has a higher protein expression level and phosphorylation, and is found in the nucleus of the MSNs of 30 weeks old R6/1 mice and 12 weeks old R6/2 mice. Elk-1 does not co-localize with mHtt, which suggests a higher transcriptional activity compared to WT mice (Roze et al., 2008a; Anglada-Huguet et al., 2012). The authors suggested that the change in Elk-1 expression may be a compensatory mechanism to protect MSN in response to mHtt-induced stress.

Elk-1 is a member of a subfamily of proteins called ternary complex factors (TCF). Elk-1 is a transcriptional activator, as it interacts with serum response factor to bind jointly to serum response elements in the promoters of several immediate-early genes (IEGs), such as c-fos and egr-1. In the CNS, Elk-1 is activated by ERKs in response to neurotrophins and neurotransmitters.

Anglada-Huguet et al. have shown that down-regulation of Elk-1 by siRNAs produces caspase 3 cleavage and cell death in immortalized HD mouse (Q111/Q111) cells, but not in wild-type cells (Anglada-Huguet et al., 2012). Thus, the induction of Elk-1 expression in HD may be considered to be a neuroprotective compensatory mechanism. However, transcriptional activity at the *c-fos* promoter was impaired in the striatum of R6/2 transgenic mice, despite activation/phosphorylation of Elk-1 (Roze et al., 2008a). As mentioned above, the reduction of MSK1 in R6/2 mice may partially impair the impact of Elk-1 activation. Elk-1 can be considered as an “inducible” striatal marker in HD, likely producing a neuroprotective self-defense mechanism. Further studies are awaited to better understand how the increase in Elk-1 plays a role in striatal degeneration at late stage in animal models of HD.

### Neutral striatal markers

#### Capucin (a.k.a. Tmem90a)

Capucin, a gene of unknown function is preferentially expressed in the striatum (de Chaldee et al., 2003). Notably, lower capucin mRNA levels have been detected in the R6/1 transgenic mouse model of HD (Desplats et al., 2006), R6/2 and in primary cultures of rat striatal neurons expressing a mutant fragment of human Htt than in the corresponding controls (de Chaldee et al., 2006). However, *in vivo* experiments showed that capucin overexpression is not able to counterbalance mHtt-induced toxicity in the striatum in a lentiviral mouse model of HD (Galvan et al., 2012b). Mice that were knockout for capucin gene had similar susceptibility to mHtt-induced toxicity as wild type age-matched littermates. Size and number of ubiquitin-containing inclusion produced by overexpression of mHtt is these mice were similar to those detected in wild type mice (Galvan et al., 2012b). Capucin downregulation in HD mouse models could be a direct consequence of the transcriptional dysfunction occurring in HD without major consequence on MSN survival. Thus, capucin may be considered as a “neutral” striatal gene.

#### Hippocalcin

Hippocalcin, a neuronal calcium sensor protein, is also known as p23k. Although the physiological role of hippocalcin is not completely understood, it is implicated in the regulation of neuronal viability and plasticity. Evidences showed that hippocalcin is important for the homeostasis of intracellular calcium levels (Amici et al., 2009). Hippocalcin can protect hippocampal neurons against excitotoxicity induced damage by enhancing Ca^2+^ extrusion and maintaining ideal intracellular Ca^2+^ levels (Masuo et al., 2007).

The decreased expression of hippocalcin in different mouse models of HD suggested a role of this protein in striatal vulnerability. Rudinskiy and collaborators studied this hypothesis in primary culture of striatal neurons (Rudinskiy et al., 2009). Hippocalcin was overexpressed using lentiviral vectors in neurons that expressed mHtt (N-terminal fragments with 82 glutamine repeat). Analysis of different outcomes related to degeneration indicated that hippocalcin was not neuroprotective. In addition, overexpression of hippocalcin did not protect neurons subjected to mitochondrial dysfunction caused by 3-nitropropionic acid or glutamate-induced excitotoxicity, two conditions inducing increase in cytoplasmic Ca^2+^ concentrations (Rudinskiy et al., 2009). Thus, hippocalcin may have deregulated expression, in absence of major consequences in neuronal survival. In this case, as capucin, hippocalcin may be seen as “neutral” striatal marker. However, it cannot be excluded that hippocalcin could have an effect in different HD models, including animal models that express full length mHtt.

### Other possible pathways to be investigated

**Figure 1:**
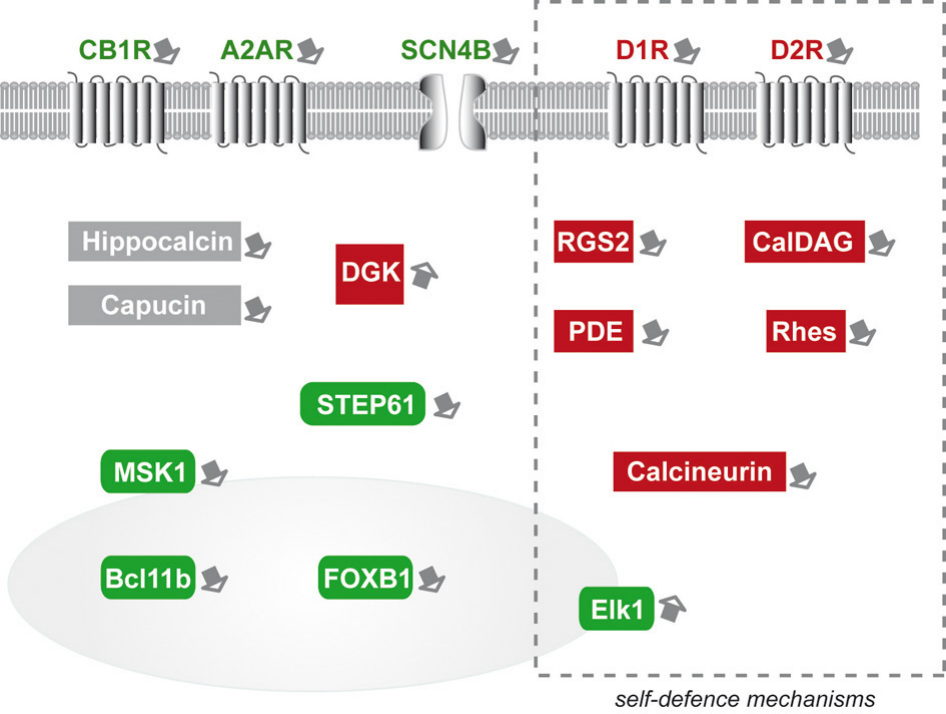
**Schematic representation of the striatal markers that have been experimentally studied as potential modifiers of mutant huntingtin toxicity in HD**. Green boxes symbolize markers that are “neuroprotective.” Red boxes symbolize markers that are “protoxic.” Expression changes in markers included in the dotted-line rectangle may represent, at least in part, self-defense-mechanisms. Markers in gray boxes would have altered expression without major consequences on mHtt. Note that striatal gene modifiers have broad biological functions and cellular localization, including neurotransmitters binding, intracellular signaling (kinases and phosphatases), and transcription activators. The nucleus is symbolized by the gray colored round form. MSK1 and Elk1 can be found in the cytoplasm and upon activation translocate in the nucleus.

Nowadays, the number of studies trying to decipher the functions of this small number of striatal genes is limited. However, these pioneering studies which tried to understand their roles with regard to mHtt toxicity provided key results indicating that possibly, they are regulators of cell survival, upstream master gene/protein networks of neuronal survival (Figure 1). In particular, deregulation of membrane receptors (D1-R, D2-R, CB1-R, A2A-R, SCN4B) involved in neurotransmission in HD could directly modulate cell survival processes through different routes (e.g., MAP Kinase pathway, regulation of PGC1-α etc.). How these different receptors act to positively or negatively regulate striatal cell survival remains to be uncovered. It is likely that, for the GPCR, their effects are related to the activation of heterotrimeric G proteins leading to increased or decreased cAMP levels but could also be mediated through other pathways such as the endocytosis/β-arrestin-mediated pathway and/or interaction of heterotrimeric subunits with transmembrane ion channels (Ritter and Hall, 2009). Increased cAMP levels may be considered neuroprotective while reduction of cAMP should be “protoxic.” It is likely that mechanisms converging on cAMP level regulation are important for opposing mHtt toxicity. Indeed PDE which reduce cAMP levels is considered to increase striatal cell vulnerability to mHtt (see below). However, it is probable that the effects of striatal membrane receptors on mHtt toxicity cannot be only explained according to their inherent capability to change cAMP levels. For example, D2-R and D1-R are thought to be coupled to different α subunits (αi/o and αs/olf, leading, when stimulated separately, to a reduction and decrease in cAMP levels respectively) (Beaulieu and Gainetdinov, 2011), but both receptors seem to increase mHtt toxicity. Downstream cAMP changes (and possible through independent mechanisms) the protoxic effects of D2-R may involve inhibition of the pro-survival kinase Akt (Marion et al., 2014) while D1-R effects may involve CDK5 (Paoletti et al., 2008). In line with these complex mechanisms, CB1-R which decrease cAMP levels when stimulated alone, are rather neuroprotective against mHtt through a mechanism that remains to be elucidated. One possibility is that co-activation of D2-R and CB1-R which increase cAMP so that the loss of CB1-R in HD may result in reduced cAMP levels and a protoxic effect which would depend on the presence of D2-R (Glass and Felder, 1997). There also exist a number of very complex cross talks between membrane receptors signaling in striatal neurons that could participate to more complex/integrated biological effects when their stimulation occurs simultaneously. In particular, receptors can heteromerize, which changes their intracellular signaling impact. For example, D1-R/D2-R heteromers act preferentially through Gαq changing signaling as compared to each receptos separately. Another interesting example is related to A2A-R/D2-R heteromers. The activation of A2A-R in these heteromers reduced the binding of dopamine on the D2-R (Ferre et al., 2008). Reciprocally, stimulation of D2-R represses the activation of adenylyl cyclase by A2-R. Other pathways may also be involved. For example, the activation of β-arrestin signaling by A2A-R/D2-R heteromers is stronger and more transient as compared to D2-R alone (Borroto-Escuela et al., 2011). In summary, the mechanisms through which those different membrane receptors act all together on mHtt toxicity (as causal factors or as key actors of compensatory/self-defense mechanisms) are largely unknown but likely involve extremely complex/integrated signaling.

Similarly cytoplasmic signaling proteins (PDE, MSK1, STEP61, DGK) can also act upstream or downstream master regulators of cell survival (CREB, MAPK/Erk1). Other striatal markers seem to be involved in molecular steps between membrane receptor signaling and downstream cytoplasmic effectors. This is the case for RGS2 and CalDAG-GEF1. Other striatal markers may not act directly on signaling processes regulating transcription or survival. Indeed, it is likely that some markers, such as Rhes, may involve key cellular “housekeeping” mechanisms such as SUMOylation of proteins and autophagy. Finally, Bcl11b and FOXP1 are good examples of striatal marker that can be directly implicated in the regulation of transcription, and the inherent state of differentiation of MSN.

The study of the role of striatal markers in striatal vulnerability in HD suggests that these gene products, likely associated with highly specific neurobiological functions (and as such they are markers of highly differentiated non-dividing cells), may be, on the one hand, the most vulnerable targets of mHtt-induced transcription deregulations and, on the other hand, key “switches” of striatal adaptive changes, that may be considered as self-defense mechanisms.

How these different striatal markers functionally interact each other remains to be precisely assessed. It is quite obvious that currently the puzzle is not complete and that many more actors are involved in the vulnerability of the striatum. Indeed, beyond the few striatal markers that have been reviewed above, many others may also act as modifiers of mHtt. When considering striatal markers with relatively stringent criteria (see paragraph above), it clearly appears that only a small proportion of striatal markers has been experimentally studied. It is beyond the scope of this review to provide extensive speculations on every striatal marker that have never been studied in the context of HD research. However, it is worth mentioning that many of them, which abnormal expression in the striatum of HD patients have been observed long ago, have never been studied for their capacity to modify mHtt toxicity. For example, neurotensin, whose expression is high in the striatum as compared to other brain regions, has been found abnormally increased in the HD striatum (Nemeroff et al., 1983). Many newly identified striatal markers have been found deregulated in HD mouse models (Brochier et al., 2008; Mazarei et al., 2010). For example, the upregulation of IDO-1 (indoleamine 2,3-dioxygenase) in YAC128 HD mice may be seen as a risk factor for striatal cells, since deletion of IDO-1 protects the striatum against excitotoxicity (Mazarei et al., 2013b). Since kynurenine pathway likely plays a role in HD pathogenesis (Thevandavakkam et al., 2010), it is possible that IDO-1 is a modifier of mHtt toxicity (Mazarei et al., 2013a). This remains to be further assessed.

## Conclusion

It is very difficult to know whether a change in expression of a given striatal marker in HD represents a compensatory mechanism, and/or a phenomenon that will contribute to striatal degeneration. This question needs to be experimentally addressed. However, all the gene products that have not yet been explored represent a pool of potential candidate modifiers of mHtt, relevant to striatal vulnerability. Our group and others are currently testing the effects of many newly identified striatal markers of unknown biological functions. Preliminary observations indicate that a majority of them are neuroprotective or protoxic modifiers of mHtt in cell and mouse models. As such, they could represent innovative therapeutic targets. Promoting the activity of the neuroprotective markers or blocking the activity of the protoxic gene products could help to slow the progression of symptoms and degeneration in HD. In addition, since a majority of these striatal markers have ill-defined neurobiological functions, research focused on these striatal gene products could be a unique opportunity to better define the molecular and functional complexity of the striatum, a brain region which is central stage in a broad spectrum of motor and cognitive functions and is likely implicated in different neurological and psychiatric illnesses.

### Conflict of interest statement

The authors declare that the research was conducted in the absence of any commercial or financial relationships that could be construed as a potential conflict of interest.
